# Field- and Angle-Dependent AC Susceptibility in Multigrain La_0.66_Sr_0.34_MnO_3_ Thin Films on YSZ(001) Substrates

**DOI:** 10.3390/ma19020331

**Published:** 2026-01-14

**Authors:** Gražina Grigaliūnaitė-Vonsevičienė, Artūras Jukna

**Affiliations:** 1 Department of Physics, Faculty of Fundamental Sciences, Vilnius Gediminas Technical University (Vilnius Tech), LT-10223 Vilnius, Lithuania; 2 Photovoltaic Technologies Laboratory, Department of Physics, Faculty of Fundamental Sciences, Vilnius Gediminas Technical University (Vilnius Tech), LT-10223 Vilnius, Lithuania

**Keywords:** polycrystalline LSMO films on YSZ, perovskite manganite, columnar grain structure, AC susceptibility

## Abstract

Experimental and numerical investigations of the alternating current (AC) susceptibility, $\chi \left(H\right)\;\sim \;dM/dH,$ examined multigrain La_0.66_Sr_0.34_MnO_3_ (LSMO) thin films (thickness *d* = 250 nm) grown by radio-frequency (RF) magnetron sputtering on lattice-mismatched yttria-stabilized zirconia YSZ(001) substrates. The films exhibit a columnar structure comprising two types of grains, with (001)- and (011)-oriented planes of a pseudocubic lattice aligned parallel to the film surface. Field- and angle-dependent AC susceptibility measurements at 78 K reveal characteristic peak- and tip-like anomalies, attributed to contributions from grains with three distinct directions of easy magnetization axes within the film plane. Numerical modeling based on the transverse susceptibility theory for single-domain ferromagnetic grains, incorporating first- and second-order anisotropy constants, corroborates the experimental findings and elucidates the role of different grain types in magnetization switching and AC susceptibility response. This study provides a quantitative determination of the three in-plane easy magnetization axes in LSMO/YSZ(001) films and clarifies their influence on the magnetization dynamics of multigrain thin films. The demonstrated control over multigrain LSMO/YSZ(001) thin films with distinct in-plane easy magnetization axes and well-characterized AC susceptibility suggests potential applications in magnetic memory, spintronic devices, and precision magnetic sensing.

## 1. Introduction

Strontium-doped lanthanum manganite, La_0.66_Sr_0.34_MnO_3_ (LSMO), exhibits the highest Curie temperature among perovskite manganites (*T*_C_ ≅ 370 K) and is of interest for spintronics devices and magnetic sensors due to grain boundary-driven low field magnetoresistance (LFMR) [1,2,3,4,5,6,7]. The highest magnetoresistance values are observed in polycrystalline materials, particularly thin films [5,6,7,8,9,10]. Although microstructural adjustment has been used to enhance LFMR, the magnetization behavior of individual grains and the regions between grains remain poorly understood [9,11,12].

The preparation and characterisation of polycrystalline LSMO films grown on polycrystalline or amorphous substrates have been reported in numerous studies [3,4,5,6,13,14]. In most cases, the films exhibit a random grain distribution, which makes modelling of their magnetic properties challenging due to varying grain size and oreintation, internal strain, and possible interactions between grains [3,5,15,16,17].

In contrast to many earlier studies, oriented LSMO films grown heteroepitaxially by RF magnetron sputtering on (100) planes of slightly lattice-mismatched cubic YSZ(001) were investigated in this work. Previous studies have shown that the as-grown LSMO/YSZ(001) films exhibit a unique multigrain structure, with coexisting grains having (001)- and (110)-oriented crystallographic planes parallel to a film surface [7,10]. These films therefore provide an interesting model system for studying magnetization reversal and switching in multigrain ferromagnetic systems.

To investigate magnetic properties, particularly field-induced magnetization switching, in multigrain LSMO/YSZ(100) films, both experimental measurements and modeling of the alternating current (AC) susceptibility, $\chi \left(H\right),$ were carried out. AC susceptibility, defined as the differential response of the sample magnetization (χ(*H*) ~ d*M*/d*H*) to a weak oscillating magnetic field *h*_AC_ (*h*_AC_ << *H*) [18,19], was analysed to evaluate the contributions of different grains. Characteristic peak- and tip-like anomalies in the χ(*H*) curves were used to assess grain-specific magnetization behaviour. By varying the angle β between *H* and *h*_AC_ within the film plane (0° ≤ β ≤ 90°), the angle dependence of the susceptibility was investigated, including the two limiting cases corresponding to the parallel $(\chi_{p}$, β = 0°) and transverse ($\chi_{t}$, β = 90°) susceptibility components of the response. These components may be related to the magnetic properties of the LSMO near the substrate surface, where the substrate interaction could strain the film. The crystallographic axes of the substrate likely determine the overall structure, and the third axis may reflect the magnetic orientation of the ferromagnetic domains in the (partially relaxed) LSMO film.

Numerical modelling of the susceptibility of ferromagnetic grains with uniaxial anisotropy and varying orientations of three easy magnetization axes within the film plane corroborated the experimental findings. The calculations followed the theory of transverse susceptibility for a single-domain ferromagnet, originally developed by A. Aharony et al. [20] and subsequently extended by other authors [21,22]. Unlike previous studies, both the first-order and second-order anisotropy constants were incorporated to model the susceptibility of individual grains and to elucidate their respective contributions to the magnetization-switching behaviour of LSMO/YSZ films.

The novelty of our work lies in determining the three easy magnetization axes within the LSMO film plane and confirming their presence in samples LSMO/YSZ(100) through numerical simulations based on the transverse susceptibility theory, with both the first-order (*K*_1_) and second-order (*K*_2_) anisotropy constants included in the modeling framework.

## 2. Characterization of Grown Films and Experimental Methods

The thin films La_0.66_Sr_0.34_MnO_3_ (LSMO), with a thickness of 250 nm, used in this study were grown in situ at 750 °C by RF magnetron sputtering on commercially available (001)-oriented cubic yttria-stabilized zirconia (YSZ) substrates, as described in Ref. [7]. Comprehensive structural characterization, performed at room temperature by X-ray diffraction (XRD) (SmartLab X-Ray diffraction, Rigaku, Tokyo, Japan), transmission electron microscopy (TEM) (Tecnai G2 F20 X-TWIN, FEI, Eindhoven, The Netherlands), and scanning electron microscopy (SEM) (Helios Nanolab 650, FEI, Amsterdam, The Netherlands), revealed a distinct columnar grain structure in the LSMO films, with an average in-plane grain diameter of approximately 50 nm.

XRD θ–2θ scans demonstrated oriented film growth within the film plane and the coexistence of grains with different crystallographic orientations, namely the (001) and (011) planes parallel to the film surface [7]. Furthermore, pole figures of the 002_LSMO_ and 011_LSMO_ reflections [7], together with TEM cross-section images, confirmed that the grains grew heteroepitaxially on the lattice-mismatched YSZ(001) substrate, following the epitaxial relationships: (001) LSMO[110]//(001) YSZ[100] and (110) LSMO[110;100]//(100) YSZ[100].

La_0.66_Sr_0.34_MnO_3_ places LSMO in the optimally doped ferromagnetic metallic regime. In this composition, the double exchange interaction between the Mn^3+^ and Mn^4+^ ions is strongest, resulting in robust ferromagnetism and high electrical conductivity. The Curie temperature is well above 300 K, ensuring that measurements at cryogenic temperatures are well within the ferromagnetic phase. This material also exhibits large and stable magnetization and well-defined magnetocrystalline anisotropy, which are essential for detecting and resolving AC susceptibility anomalies associated with magnetization switching.

**Figure 1:**
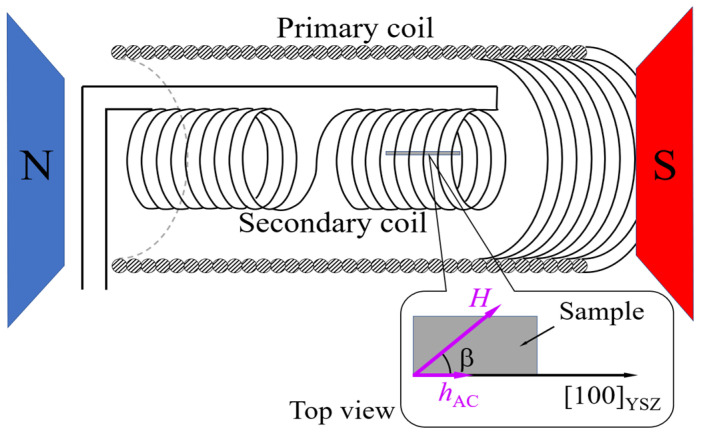
Schematic diagram of the experimental setup for measuring field-dependent AC susceptibility. Measurements cover various in-plane angles β between the DC field *H* and the alternating field *h*_AC_, aligned parallel to the long axis of the film, as illustrated in the inset. Here, N and S denote the north and south poles of the magnet generating the DC magnetic field *H*, and *h*_AC_ indicates the direction of the AC magnetic field with respect to the YSZ[100] substrate crystalline direction.

The field-dependent AC susceptibility of the samples, χ(*H*) ~ d*M*/d*H*, was measured using a custom-built susceptometer (the susceptometer was designed using the laboratory facilities of Vilnius Gediminas Technical University (Vilnius Tech), Vilnius, Lithuania) (Figure 1), as described in Refs. [7,23]. The susceptometer consisted of two identical coil sections wound in opposite directions and connected in series to produce a zero-output signal in the absence of a sample [23]. Stripe-shaped LSMO films, each measuring 5.0 × 10.0 mm^2^ were prepared for the susceptibility measurements.

During the measurements, the LSMO film remained fixed while an AC magnetic field, *h*_AC_ ≈ 0.1 kA/m, was applied along its longitudinal axis. The study explores AC susceptibility at several fixed in-plane angles between the DC magnetic field *H* and *h*_AC_. The DC field swept from −300 kA/m to +300 kA/m, achieving full negative and positive magnetization *M* saturation in the sample. The voltage induced in the secondary coil was detected within a narrow frequency band [24,25], specifically at the fundamental frequency (*f* = 47 kHz) using a lock-in amplifier (type 232B nanovoltmeter, Unipan, Warsaw, Poland).

The measured signal, arising from the time-dependent magnetization *M* of the sample positioned in one section of the pickup coil, can be expressed [25,26] as:

(1) $$V_{AC}\left(H,t\right)=f\xi V\frac{dM}{dH}h_{AC}\sin\left(2\pi ft\right).$$

Here, *V* denotes the volume of the tested LSMO sample.

Magnetization measurements of the LSMO films at 78 K carry an estimated uncertainty below 10%, resulting from instrumental limitations and sample handling, which provides sufficient accuracy for analyzing the magnetic properties.

## 3. Results

### 3.1. Field-Dependent AC Susceptibility Measurements

**Figure 2:**
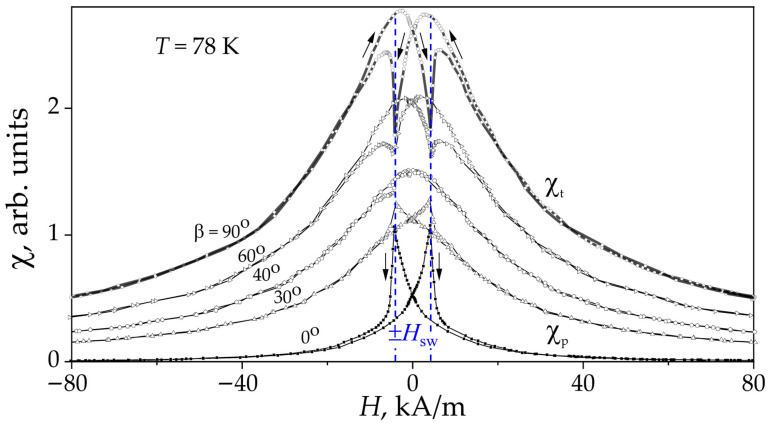
Field-dependent AC susceptibility, χ(*H*), measured at 78 K for a 250 nm thick multigrain LSMO/YSZ(100) film (5.0 × 10.0 mm^2^). The DC magnetic field *H* was applied at in-plane angles β *=* 0°, 30°, 40°, 60° and 90° relative to the AC excitation field *h*_AC_, aligned parallel to the long axis of the film (inset, Figure 1). Arrows indicate the sweep direction of the field. Two symmetric peaks in the parallel component of susceptibility $\left(\chi_{p}\right)$ correspond to magnetization switching at *H* = ±*H*_sw_, while the transverse component susceptibility $\left(\chi_{t}\right)$ exhibits broad peaks and tip-like anomalies with amplitudes that vary depending on β. The dashed blue lines are a guide for the eye.

The field-dependent susceptibility of the films, χ(*H*), was measured at 78 K for five in-plane angles between *H* and *h*_AC_ (β *=* 0°, 30°, 40°, 60°, and 90°) under bipolar field sweeping, as shown in Figure 2. During measurements, *H* varied from high positive to negative values and back to positive, achieving full positive or negative saturation of the film’s magnetic moment at *H* > 300 kA/m. The commonly used field configurations, *H* ‖ *h*_AC_ (β *=* 0°) and *H* ⊥ *h*_AC_ (β *=* 90°), correspond to the parallel, $\chi_{p}$(*H*), and transverse, $\chi_{t}$(*H*), susceptibilities, respectively.

Two well-defined, symmetrical peaks in the bipolar plots (Figure 2) indicate typical magnetization switching behavior in the films at *H* = ±*H*_sw_ (*H*_sw_ *≈* 4.0 kA/m). The transverse susceptibility $\chi_{t}$(*H*), however, shows unusual field-dependent behavior, displaying two broad peaks accompanied by two sharp, tip-like anomalies at *H* = ±*H*_sw_. In contrast, the susceptibility measured at β *=* 40° exhibits a single broad peak near *H* ≈ 0 kA/m and shows almost no discernible anomalies at *H*_sw_.

Figure 2 shows that the amplitude of the peak-like anomaly decreases as β increases from 0° to 40°, while the amplitude of the tip-like anomaly rises as β increases from 40° to 90°. The critical fields corresponding to both peak-like and tip-like anomalies remain nearly constant with changes in the in-plane angle between *H* and *h*_AC_.

The observed angular dependence of the AC susceptibility (Figure 2) demonstrates that the magnetization dynamics in multigrain LSMO/YSZ(001) films is governed by grains with distinct easy-axis orientations. The peak- and tip-shaped features in the transverse response reflect the interaction between these grain populations and their respective anisotropy fields. The stability of the switching fields across all measured orientations indicates robust magnetic anisotropy, confirming that the magnetization reversal process is dominated by grain-specific switching mechanisms.

Several studies [24,25] have reported complex field-dependent transverse susceptibility in ferromagnetic systems. Theoretical models describing transverse susceptibility in polycrystalline materials with randomly oriented ferromagnetic grains were first developed by Aharoni et al. [20] and later extended by other researchers [27]. The generalized theory for multiparticle systems predicts the appearance of three distinct peaks, rather than sharp tip-like features, in unipolar field scans from negative to positive saturation. Two of these peaks appear symmetrically in the anisotropy field (*H* = ±*H*_A_), while the third peak, located near the switching field (*H* = ±*H*_sw_), is broadened due to variations in grain orientation, size distribution, and internal strain. Experimental evidence of tip-like anomalies in the transverse susceptibility curves has been reported for various multiparticle ferromagnetic systems [27,28,29,30], although the precise microscopic origin of these anomalies is not fully understood.

Clearly defined pit-like anomalies in the transverse biased susceptibility have previously been observed in our studies of multigrain LSMO films grown on lattice-mismatched YSZ(001) substrates. The appearance of these anomalies in the $\chi_{t}$(*H*) dependence was interpreted within the framework of the transverse susceptibility theory for isolated uniaxial ferromagnetic particles, assuming the coexistence of grains with several fixed in-plane easy-axis orientations [7]. The primary focus was on the comparative analysis of characteristic anomalies in field-dependent magnetoresistance and AC susceptibility for both in-plane and out-of-plane magnetic field geometries.

In the present study, attention is directed toward AC susceptibility measurements with the external field *H* applied within the film plane at various angles relative to the AC field *h*_AC_. The contribution of grains with different easy-axis orientations relative to the applied field is analyzed in detail to elucidate the origin of the angle-dependent anomalies observed in the susceptibility data. The transverse susceptibility model is therefore extended to describe multigrain LSMO films characterized by the coexistence of grains with distinct crystallographic orientations and corresponding variations in their preferred magnetization directions.

### 3.2. Modelling of χ(H) Angular Dependencies

The crystallographic orientation of individual grains and, in particular, the orientation of their easy magnetization axes within the film plane, plays a key role in modeling the magnetization and switching behavior of multigrain LSMO films. Structural studies have confirmed the oriented growth and coexistence of grains with (100) and (110) oriented crystallographic planes parallel to the film surface in LSMO films deposited on lattice-mismatched YSZ(100).

Despite the significant lattice mismatch between LSMO and YSZ (*a*_YSZ_ ≅ 0.514 nm, *a*_LSMO_ ≅ 0.387 nm), heteroepitaxial growth occurs due to the approximate lattice relationships *a*_YSZ_ ≅ $\sqrt{2}$ *a*_LSMO_ and 3 *a*_YSZ_ ≅ 4 *a*_LSMO_. For the (100)-oriented grains, a cube-on-cube growth mode with a 45° in-plane rotation satisfying the epitaxial relationship (001)LSMO[110]//(001)YSZ[100] has been identified. On the contrary, the (110)-oriented LSMO grains follow a different epitaxial relation, namely (110)LSMO[110;100]//(100)YSZ[100].

Assuming that the [100] (or equivalently [010]) crystallographic direction corresponds to the preferred easy magnetization axis of LSMO (110), two in-plane easy-axis orientations are available for the (110)-oriented grains, along the mutual perpendicular direction. The (110) grains with their easy axes aligned parallel and perpendicular to the long axis of the films ([100]_YSZ_-axis in the inset of Figure 1) are denoted as G(0) and G(90), respectively. Meanwhile, the (100)-oriented grains, whose [100] crystallographic axis (and thus their easy magnetization direction) is rotated by 45° relative to the long axis of the film, are denoted as G(45).

In the following analysis, each grain is treated as a single-domain ferromagnetic particle with uniform magnetization, justified by the average grain diameter (~50 nm), which remains below the threshold for multidomain formation (typically observed for grains with *d* ≥ 100 nm). To evaluate the contributions of the three grain populations, G(0), G(45), and G(90), characterized by distinct easy-axis orientations (0°, 45°, and 90°, respectively) to the overall magnetization, AC susceptibility, and field-induced switching behavior, the total energy density of a uniaxial single-domain grain is expressed as the sum of two terms: the anisotropy energy and the Zeman energy [27]:

(2) $${E\;(\theta }_{M})={sin}^{2}\left(\theta_{M}-\theta_{K}\right)-2h\;cos\left(\theta_{M}-\theta_{H}\right).$$

Here, $\theta_{M},\;\theta_{K},\;{and\;\theta }_{H}$ denote the polar angles of the magnetization vector *M*, easy-axis *K*_ea_, and the applied magnetic field *H*, respectively. The dimensionless magnetic field is defined as $h=\mu_{0}M_{sat}H/2K_{1}$, where *K*_1_ is the uniaxial anisotropy constant, and $M_{sat}$ is the saturation magnetization. This approach allows identification of the stable and metastable magnetization orientations and provides insight into the switching behavior and AC susceptibility response of the multigrain LSMO films.

The equilibrium angle of the magnetization vector (θ_M0_) as a function of the applied field *h* can be determined numerically from Equation (2) by minimizing the total energy. Characteristic switching fields are identified by simultaneously setting the first and second derivatives of the energy with respect to θ to zero.

**Figure 3:**
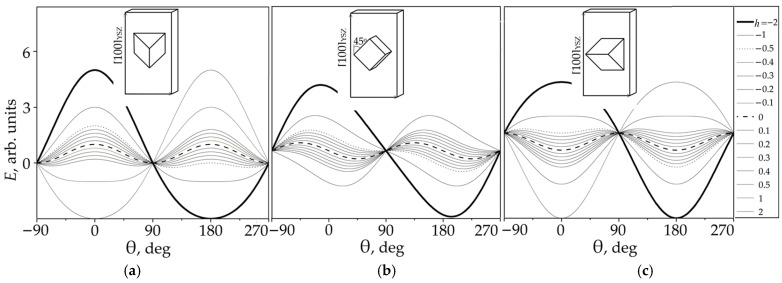
Numerically calculated energy *E* as a function of the angle of the magnetization vector *M*, based on Equation (2), for grain populations G(0) (**a**), G(45) (**b**), and G(90) (**c**) with easy-axis angles θ_K_ = 0°, 45°, and 90°, respectively, at several fixed values of the applied dimensionless AC magnetic field −2 ≤ *h* ≤ 2.

Figure 3a–c presents the numerically calculated energy profiles *E*(θ) for the G(0), G(45), and G(90) grain populations, corresponding to polar angles of their easy magnetization axes θ_K_ = 0°, 45°, and 90°, respectively.

The observed energy landscapes provide insight into the mechanisms of field-induced rotation and switching of the magnetization vector for different grain orientations. Analysis of the evolution of local energy minima as the reduced field *h* varies from positive to negative saturation reveals distinct behaviors for the three types of grains. For the G(0) grain (Figure 3a), the equilibrium magnetization angle remains fixed at θ_MO_ = 0° for all positive *h* values and switches abruptly to 180° at *h* = −1. A similar switching process occurs for the grain G(45) (Figure 3b), but with a lower field value of approximately *h* = −0.5. In contrast, the grain G(90) (Figure 3c) exhibits a smooth and reversible rotation of *M*_0_ as *h* changes from positive to negative saturation, without abrupt change.

Taking into account different switching behavior of the grains G(0), G(45), and G(90), the contribution of each grain type to the transverse susceptibility of the films is analyzed. Transverse susceptibility $\chi_{t}\left(h\right)$, which describes the magnetization response to a small alternating magnetic field *h*_AC_ applied along the [100]YSZ-axis (inset of Figure 1), can be calculated using the following expression [28]:

(3) $$\chi_{t}\left(h,\;\theta_{K}\right)=\frac{3}{2}\chi_{0}\frac{{cos}^{2}\theta_{M0}}{F\left(h,\theta_{M0},\theta_{K}\right)}.$$

Here, $\chi_{0}=M^{2}/3K_{1}$, the $\theta_{M0}$ and $\theta_{K}$ are the equilibrium angle of the magnetization vector and the polar angle of the easy-axis *K*_ea_ at a given applied field, both determined by minimizing the total energy. $F\left(h,\theta_{M0},\theta_{K}\right)$ is the denominator function representing the second derivative of the free energy:

(4) $$F\left(h,\theta_{M0},\theta_{K}\right)=\frac{\partial^{2}E}{\partial \theta_{M}^{2}}=hcos\theta_{M}+cos2\left(\theta_{M}-\theta_{K}\right).$$

**Figure 4:**
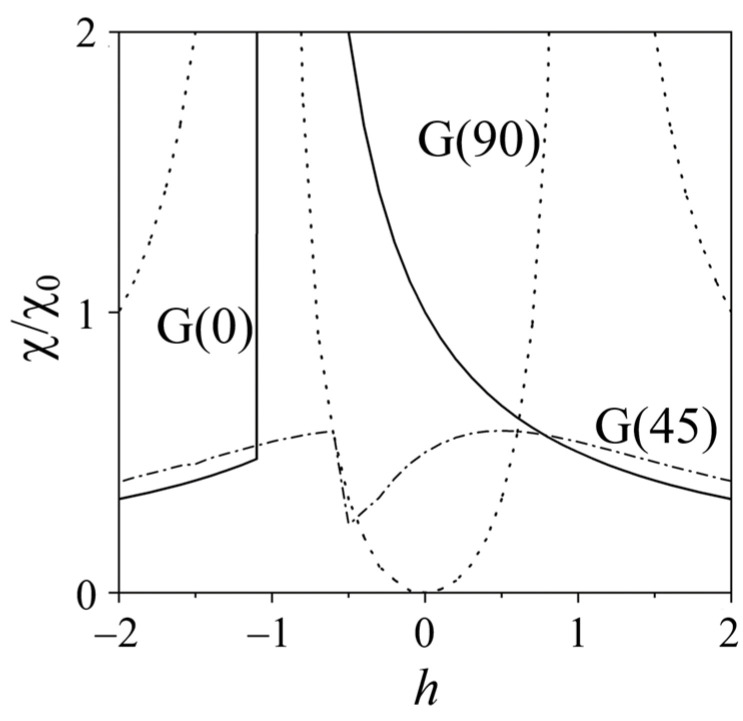
Transverse susceptibility $\left(\chi /\chi_{0}\right)$ as a function of the dimensionless field *h*, calculated for the grains G(0), G(45), and G(90). The alternating field *h*_AC_ is applied along [100]YSZ-axis (inset of Figure 1) and perpendicular to the static magnetic field *H*.

Figure 4 shows the numerically calculated $\chi_{t}\left(h,\;\theta_{K}\right)⁄\chi_{0}$ plots for the grains G(0), G(45), and G(90), with a dimensionless field varying over the interval −2 ≤ *h* ≤ 2. The clearly defined pit-like anomaly observed in the $\chi_{t}\left(h\right)$ curve of G(45) at *h* = −0.5 can be explained by considering that the magnetization vector *M* of the grain just before switching aligns nearly parallel to the applied AC field (see Figure 3b). In this configuration, the small AC field applied along *M*_0_ cannot significantly rotate the magnetization and as a result, $\chi_{t}$ reaches a minimum when $\theta_{M0}$ approaches 90°.

Two symmetrical peaks observed in the $\chi /\chi_{0}\left(h\right)$ plot of the grain G(90) at *h* = ±1.0 correspond to the anisotropy field of the material *H*_A_ = 2*K*_1_/*M*_Sat_, according to the prediction in Ref. [20]. In contrast, sweeping the magnetic field from positive to negative in the grain G(0) produces a single peak in the plot of $\chi /\chi_{0}\left(h\right)$ at *h* = −0.5 (Figure 4), reflecting a change in magnetization.

Our calculations use the Aharoni model [20] for uniaxial crystals characterized by a first-order anisotropy constant (*K*_1_). To gain additional insight into the origin of angle-dependent transverse susceptibility anomalies, this work proposes introducing the second-order anisotropy constant (*K*_2_) in the expression for free energy [29] as follows:

(5) $$F\left(h,\theta_{M0},\theta_{K}\right)=hcos\;\theta_{M}+cos2\left(\theta_{M}-\theta_{K}\right)+k_{2}(cos2\left(\theta_{M}-\theta_{K}\right)-cos4\left(\theta_{M}-\theta_{K}\right)).$$

Here, $\theta_{M},\;\theta_{K},\;{and\;\theta }_{H}$ denote the polar angles of the magnetization vector *M*, easy-axis *K*_ea_, and the applied magnetic field *H*, respectively, and $k_{2}=K_{2}/K_{1}$. Depending on the value of $k_{2}$ the anisotropy-energy ground state may change from an easy-axis configuration $K_{1}>0$ to an easy-plane configuration $K_{1}<0$ or even to a cone state. The introduction of a modified free energy expression can create a more complex energy landscape, which produces additional equilibrium positions of the magnetization vector.

**Figure 5:**
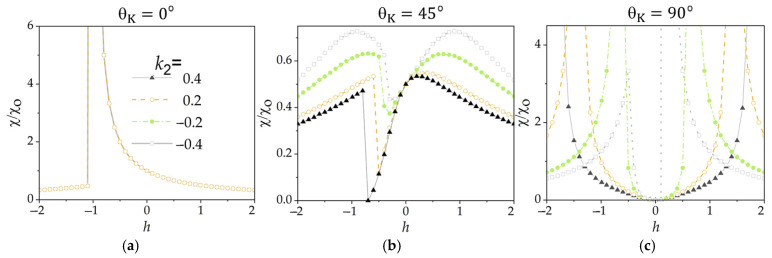
Transverse susceptibility of grains with three different easy-axis orientations ($\theta_{K}$ = 0° (**a**), 45° (**b**), and 90° (**c**)), calculated for several values of $k_{2}=\frac{K_{2}}{K_{1}}$ values, where *K*_1_ and *K*_2_ are the magnetic anisotropy constants. The AC field *h* was aligned perpendicular to the DC field *H*.

Figure 5a–c show the transverse susceptibility as a function of dimensionless *h*, calculated numerically for grains with three fixed easy magnetization directions (θ_K_ = 0°, 45°, and 90°, respectively) using four different $k_{2}=K_{2}/K_{1}$ values. These results reveal new features in $\chi_{t}\left(h\right)$ plots compared with those in Figure 4. For grain G(0) (Figure 5a), the $\chi_{t}\left(h\right)$ dependence exhibits a clearly defined switching event at *h* = −1 and this behavior remains independent of the value *k*_2_ in the range of −0.4 to + 0.4. The inclusion of the higher-order anisotropy constant improves the visibility of the jump in $\chi \left(H\right)$ at *h* = −1, highlighting its potential usefulness for detecting such switching events [31,32].

Reversible $\chi_{t}\left(h\right)$ plots exhibiting pronounced peaks are observed in Figure 5c for the grain G(90) with $\theta_{K}=90^{{}^{\circ}}$ at several *k*_2_ values (*k*_2_ = 0.4, 0.2, and −0.2). It is important to note the significant shift in the peak positions toward the lower *h* values (from 2.0 to approximately 0.7) as *k*_2_ decreases from 0.4 to −0.2, as well as the strong asymmetry in the peak amplitudes and positions of *k*_2_ = −0.4.

Meanwhile, clearly defined pit-shaped anomalies appear in the $\chi_{t}\left(h\right)$ plots shown in Figure 5b for the grain G(45). As seen in this figure, the amplitudes of these anomalies decrease and the corresponding switching fields shift markedly (from approximately −0.8 to −0.4) as *k*_2_ decreases from 0.4 to −0.4.

Thus, the inclusion of the second-order anisotropy constant *K*_2_ produces distinct angle-dependent changes in the transverse susceptibility, causing peak shifts and asymmetries in grain G(90) and pit-like anomaly shifts in grain G(45), while leaving the switching event in grain G(0) robust. In general, *K*_2_ introduces new features in $\chi_{t}\left(h\right)$ that reflect a more complex magnetization energy landscape.

To place these model-derived trends in the context of the experimental system, it is necessary to consider the structural and magnetic complexity of the films. The LSMO films exhibit a dense columnar multigrain microstructure, in which intergrain exchange interactions, dipolar coupling, strain gradients, and grain-boundary effects can influence the magnetization dynamics and the transverse susceptibility response. Accordingly, such effects are probably present to some extent in the experimental results (Figure 2). Nevertheless, the present work adopts a simplified modeling framework based on an ensemble of isolated, single-domain grains with uniaxial magnetocrystalline anisotropy to isolate and emphasize the primary role of magnetocrystalline anisotropy and grain orientation in shaping the transverse susceptibility features. Within this framework, grains with different easy-axis orientations can account for the observed susceptibility characteristics, including the peaks, pits, and their angular evolution.

## 4. Discussion

Field-dependent susceptibility anomalies were measured experimentally in the LSMO/YSZ(100) film (Figure 2), including sharp switching peaks, symmetric rounded maxima, and a distinct pit-like characteristic. These features can be interpreted by comparing the experimental behavior with results of simulation using a simplified magnetization rotation model for grains possessing different orientations of the easy magnetization axis [27,28]. Sharp peak-type anomalies originate predominantly from grains whose easy axis is aligned parallel to the applied magnetic field. Such grains are expected to exhibit strong hysteresis and well-defined switching events, since the magnetization reverses abruptly when the applied field reaches the switching threshold characteristic of single-domain ferromagnetic particles.

The rounded and pit-shaped anomalies (Figure 2) can arise from grains with oblique or perpendicular easy-axis orientations, whose transverse susceptibility responses are intrinsically smoother and more complex due to the competition between anisotropy (i.e., anisotropy energy favors magnetization pointing along certain preferred crystallographic direction) and Zeeman (i.e., Zeeman energy favors magnetization aligning with the externally applied magnetic field) energies. Together, these features reflect the angular distribution of magnetic grains within the film and the corresponding diversity of their magnetization reversal processes [33].

As proposed by the model in Ref. [20], the extremely high amplitude of the peaks can be understood through the theory of transverse susceptibility for a single isolated grain, particularly when the second derivative of the free energy *F* is set to zero in the relevant Equation (3). In real multigrain systems (Figure 2), the peak amplitudes are expected to be significantly lower, due to grain-to-grain interactions, internal strains, and nonuniform magnetization.

The calculation results for grain G(45), shown in Figure 4 (dashed-dot), help explain the origin of tip-like anomalies arising from the presence of grains G(45) in polycrystalline LSMO/YSZ(100) films. In this context, the uniaxial magnetic anisotropy predicts that the tip features should appear at lower field values (*h* = ±0.5) than the peaks (*h* = ±1.0) observed in the $\chi_{t}$(*H*) plots for grains G(0) and G(90). However, the experimental $\chi_{t}$(*H*) curves in Figure 2 indicate that all anomalies, including tips as well as sharp and rounded peaks, were found to occur at almost the same field value, *H* = *H*_sw_ ≈ ±4 kA/m.

As shown in Figure 5b, the inclusion of a second-order anisotropy constant (*K*_2_ > 0) in the free energy expression leads to a significant increase in the characteristic *h* value for the grain G(45). However, it was observed in this work that introducing positive *K*_2_ values shifts the anisotropy peaks to higher field values for the grain G(90), while the characteristic *h* values for the grain G(90) remained essentially unchanged.

In the experiments, the measured grains corresponded more closely to G(40) rather than the simulated G(45), which may partly explain the deviation between theoretical predictions and experimental observations. Such discrepancies could arise from slight variations in grain orientation, as well as additional factors not captured in the single-domain, noninteracting grain model. For example, the magnetron sputtering process used to deposit the LSMO film may induce nonuniform magnetization or nucleate small ferromagnetic domains in the underlying YSZ substrate [34], which can influence the switching behavior of the grains.

Therefore, the occurrence of characteristic peaks and tip-like anomalies in the $\chi_{t}$(*H*) plots of multigrain LSMO/YSZ(100) films at nearly the same field *H* ≈ ±4 kA/m cannot be fully explained by the magnetization rotation model for isolated single-domain particles with uniaxial anisotropy (*K*_1_) or modified with second-order anisotropy (*K*_2_). Current experimental results suggest that the model based on noninteracting grains should be refined to better represent the real structure of the multigrain LSMO films, including possible nonuniform magnetization within grains. Moreover, calculations indicate that even a small deviation of the easy axis direction 0° to 5° can significantly reduce the peak amplitudes and the *h* values. To achieve closer agreement between experimental and simulated results, one should consider correlated magnetization effects between neighboring grains, the potential formation of closed magnetization loops at zero applied field, and correlated switching within the multigrain ferromagnetic system. These factors are likely responsible for the experimental deviations observed in the G(40) grains relative to the idealized G(45) model.

The present work employs a simplified modeling framework of isolated, single-domain grains with uniaxial magnetocrystalline anisotropy to highlight the dominant role of anisotropy and grain orientation in shaping the transverse susceptibility. Within this approach, variations in the orientation of the easy magnetization axis provide a qualitative explanation for the observed susceptibility features and their angular dependence.

## 5. Conclusions

Transverse susceptibility measurements of LSMO/YSZ(100) films reveal sharp peaks, rounded maxima, and tip-like features, reflecting the diversity of grain orientations and magnetization reversal processes. Sharp peaks arise from grains with easy axes aligned to the applied field, producing abrupt single-domain switching, while rounded and tip-like anomalies originate from oblique or perpendicular grains, where competition between anisotropy and Zeeman energies leads to smoother responses.

Simulations using a simplified magnetization rotation model capture some qualitative features, but cannot fully explain that all anomalies occur at nearly the same field *H*_sw_ ≈ ±4 kA/m. Deviations between experiment and theory are attributed to experimental grains corresponding to G(40) rather than G(45), as well as effects of nonuniform magnetization, grain-to-grain interactions, and substrate-induced ferromagnetic domains. Refinement of the model to include small deviations from the orientation of the easy axis orientation, correlated magnetization, and collective switching is necessary for better agreement.

The observed trends should be interpreted in light of the structural and magnetic complexity of the films. While various microstructural effects can influence the magnetization response, a simplified model focusing on the dominant anisotropy and grain orientation captures the main features of the susceptibility, including the characteristic peaks and anomalies. These results highlight the complex interplay between grain orientation, anisotropy, and intergrain interactions in multigrain LSMO/YSZ films and provide guidance for empirically proven theoretical modeling of polycrystalline ferromagnets.

## Data Availability

The original contributions presented in this study are included in the article. Further inquiries can be directed to the corresponding authors.

## Abbreviations

The following abbreviations are used in this manuscript:

χ	Magnetic susceptibility
$\chi_{p}$	Parallel magnetic susceptibility
$\chi_{t}$	Transverse magnetic susceptibility
$\theta_{H}$	Polar angle of applied magnetic field
$\theta_{K}$	Polar angle of easy axis
$\theta_{M}$	Polar angle of magnetization vector *M*
$\theta_{M0}$	Equilibrium magnetization angle obtained by minimizing the free energy
ξ	Coupling constant
AC	Alternating current
DC	Direct-current
*E*	Total energy of a single domain
$F\left(h,\theta_{M0},\theta_{K}\right)$	The function representing the second derivative of the free energy
G(0)	Grains with an easy magnetization axis 0°
G(45)	Grains with an easy magnetization axis 45°
G(90)	Grains with an easy magnetization axis 90°
*H*	DC magnetic field
$H_{SW}$	Switching field
$h_{AC}$	Driving AC magnetic field
$K_{1}$	First-order anisotropy constant
$K_{2}$	Second-order anisotropy constant
